# Extradural cryptococcoma at the sacral spine without bone involvement in an immunocompetent patient

**DOI:** 10.1007/s00776-013-0413-2

**Published:** 2013-05-28

**Authors:** Yumiko Asanuma, Hajime Fujimoto, Hiroshi Nakabayashi, Koji Akeda, Kunihiro Asanuma, Masaru Tanaka, Takeshi Nagakura, Yoshihiro Miura, Takahiro Iino, Kunikazu Ogawa, Yuichi Kasai, Akihiro Sudo

**Affiliations:** 1 Department of Orthopedic Surgery, Mie Chuo Medical Center, 2158-5 Hisai Myojincho, Tsu, Mie 514-1101 Japan; 2 Department of Respiratory Medicine, Mie Chuo Medical Center, Tsu, Japan; 3 Department of Pathology, Mie Chuo Medical Center, Tsu, Japan; 4 Department of Orthopedic Surgery, Mie University, Tsu, Japan

## Introduction

*Cryptococcus neoformans* is an encapsulated yeast-like fungus found in avian excreta, soil, and decayed wood [1]. The respiratory tract is the primary route of cryptococcal infection from yeast inhalation, and the infection can disseminate to most other organs [2, 3], although the main target organs are the lungs and the central nervous system (CNS) [4]. The most common manifestations in cases of CNS involvement are meningitis and meningoencephalitis [5, 6]. An extradural lesion of cryptococcal infection in the CNS is a rare entity that is usually accompanied by osteomyelitis [7–14]. Cryptococcal infection is mostly found in immunocompromised patients [2, 4, 5]. However, up to 10–40 % of reported cases have been in immunocompetent individuals [5, 6, 15, 16].

An extremely rare case of isolated extradural cryptococcoma at the sacral spine without a contiguous bone lesion in an immunocompetent man is described. To the best of our knowledge, this is the first description of extradural cryptococcoma without a bone lesion.

## Case report

A 52-year-old man presented with nocturia and perianal pain 3 years before he visited our hospital. The pain increased progressively, with subsequent spread to his right leg 1 year ago. He was referred for examination in our hospital in 2010. Physical examination revealed absence of the right Achilles tendon reflex, sensory disturbance in his right S2 area, and motor palsies involving his right flexor hallucis longus and right flexor digitorum longus. The straight leg raising test was positive in the right lower extremity (60 degrees). On past history, he underwent appendectomy and tonsillectomy at the age of 17 years, when, for the first time, an abnormally high white blood cell (WBC) count was noted. Since then, he had a high WBC count on every checkup examination, and no abnormalities were seen on bone marrow aspiration at the age of 47 years. The family history revealed nothing of note.

**Fig. 1:**
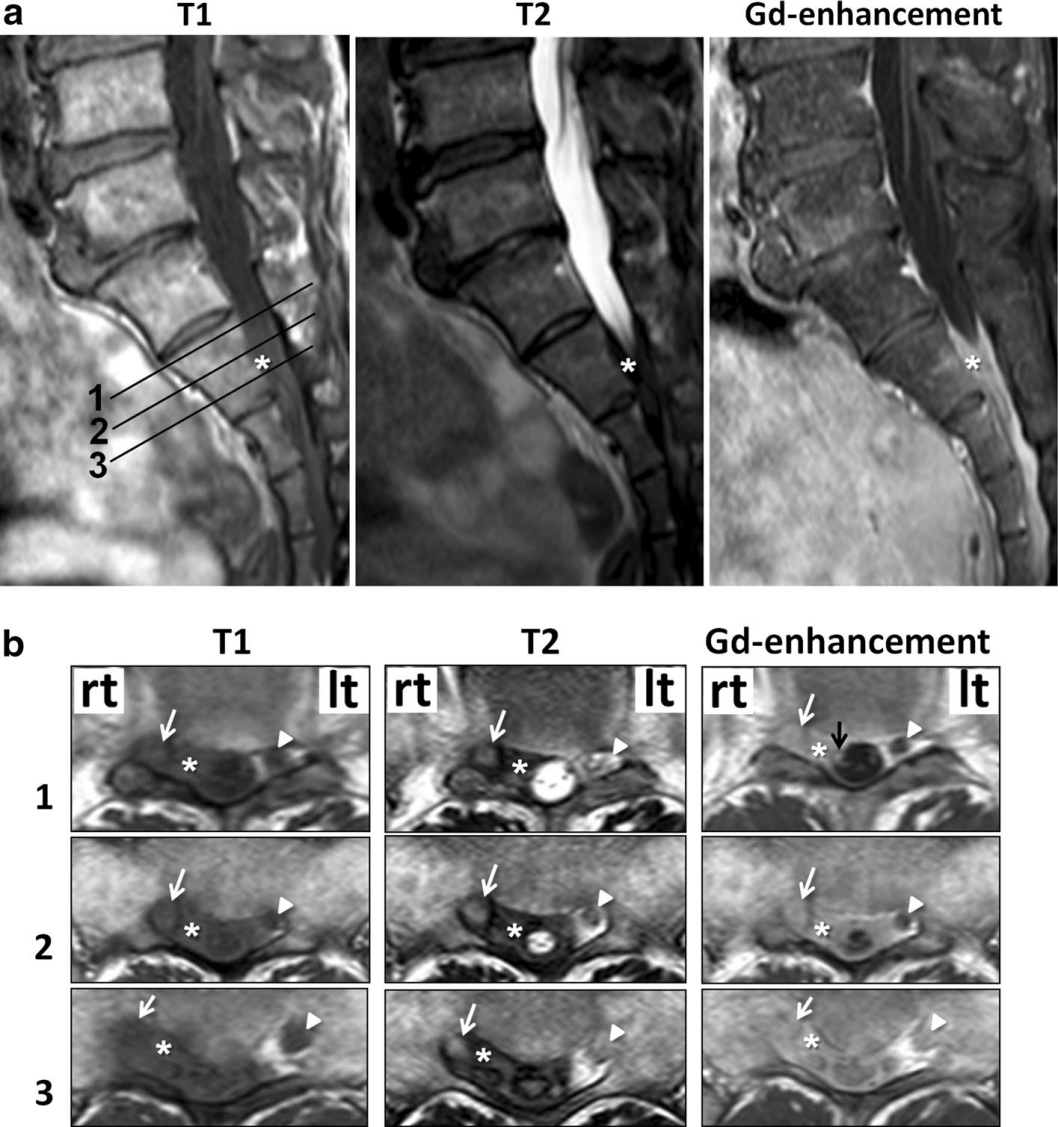
The *numbers* in the sagittal T1-weighted MRI (**a)** indicate the slice levels in the axial images (**b)**. *Asterisks* a space occupying lesion (SOL). *White arrows* (*right*) and *arrow heads* (*left*), S2 nerve root. *Black arrow* slightly enhanced cauda equina

**Fig. 2:**
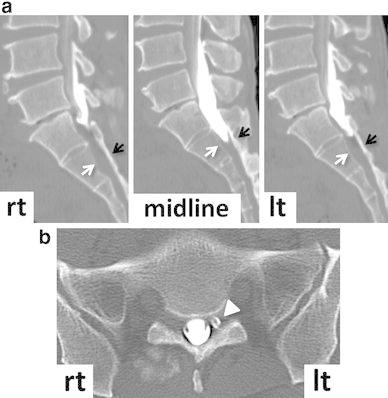
A bone lesion is not seen on sagittal (**a)** and axial (**b)** CT myelography. *White* (posterior wall of the vertebral body) and *black* (lamina) *arrows* indicate the bone without lesion (**a)**. *Arrow head* indicates left S2 nerve root (**b)**

There were no significant findings on routine blood tests, tumor markers, urine tests, and chest X-ray examinations, except for an increased WBC count (11,800/μl) with elevated neutrophils (73.2 %) and decreased lymphocytes (20.6 %), with no increase in C-reactive protein levels. On X-ray examination, the lumbosacral spine revealed no abnormalities. Magnetic resonance imaging (MRI) of the spine showed a space occupying lesion (SOL) with low intensity on both T1- and T2-weighted imaging examinations in the extradural space at the S2 level, which encircled the dural sac and the right S2 nerve root (Fig. 1). The extradural SOL, the right S2 nerve root, and the cauda equina in the dural sac were slightly enhanced by gadolinium. The left S2 nerve root, which was not surrounded by the SOL, showed no change in intensity on both T1- and T2-weighted imaging and gadolinium enhancement. No change in intensity was seen in the contiguous bone of the extradural SOL on the sagittal MRI images (Fig. 1a). No bone destruction was seen on sagittal computed tomography (CT) (Fig. 2a). CT myelography indicated that cerebrospinal fluid (CSF) flow was blocked at the S1 level on the right side, although CSF flow was seen at the S1/S2 intervertebral disc level on the left side (Fig. 2a). The right S2 nerve root was not detected, although the left S2 nerve root appeared normal on axial CT myelography (Fig. 2b). CSF analysis showed clear, colorless fluid with a slightly elevated protein level (64 mg/dl) and normal ranges of cell count (5 cells/μl of lymphocytes), glucose (57 mg/dl), and chloride (125 mEq/l).

**Fig. 3:**
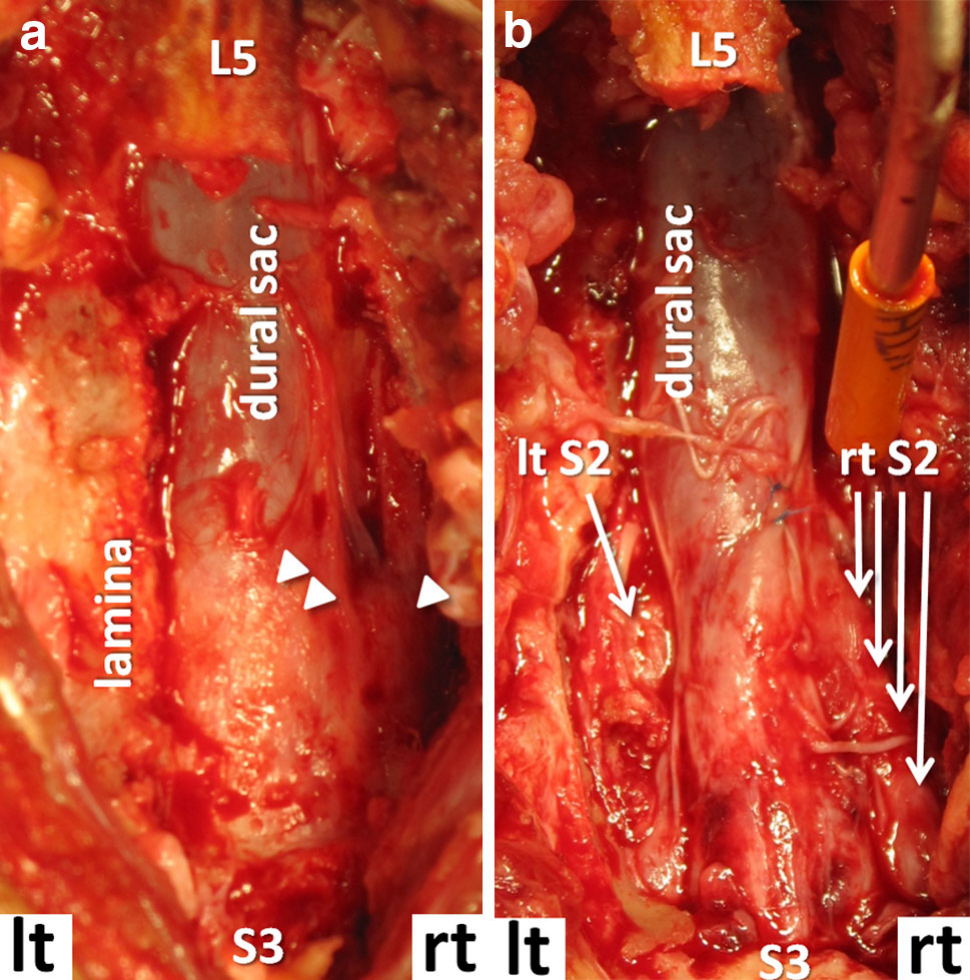
Photographs taken after laminectomies of S1 and S2 (**a)** and after removal of the SOL (**b)**. The fibrous lesion presents yellowish-white and densely adherent to the right S2 nerve root (*one arrow head*) and the dural sac (*two arrow heads*) (**a**)

The patient underwent laminectomy from the S1 to S2 levels through a posterior approach (Fig. 3). The paraspinal muscles and the laminae appeared normal. After laminectomy, a yellowish-white, thick, fibrous lesion was exposed in the extradural space, which did not adhere to the laminae. However, since the fibrous lesion was densely adherent to the dura mater and the right S2 nerve root, it was impossible to remove completely, although the dural sac and the right S2 nerve root were decompressed. There was neither abscess formation nor a tumor-like mass in the extradural space. The left S2 nerve root was easy to expose, since there was no fibrous lesion around it. The pain in his right lower extremity and perianal area significantly decreased after surgery.

**Fig. 4:**
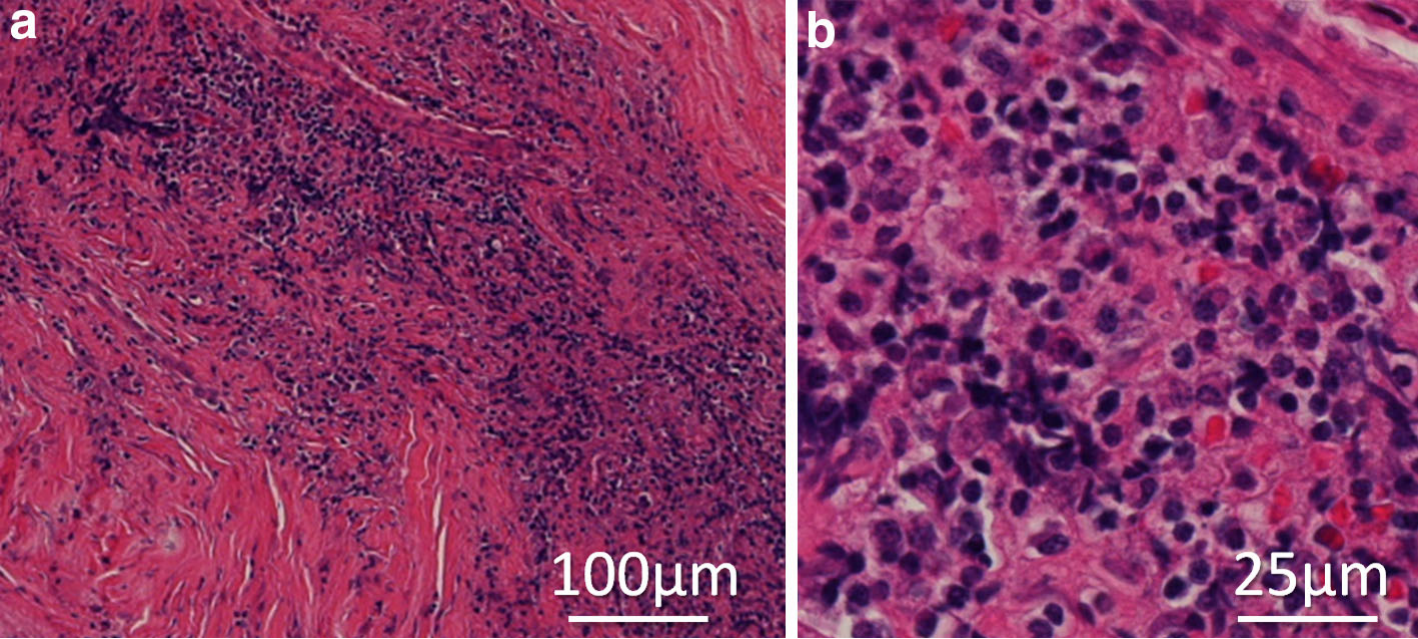
The histological examination of the extradural mass lesion harvested during surgery by hematoxylin-eosin (HE) staining. Granulomatous inflammation, accompanied by numerous inflammatory cells, is seen (**a**). The predominant cell type in the inflammatory area is lymphocytes on high magnification (**b**)

**Fig. 5:**
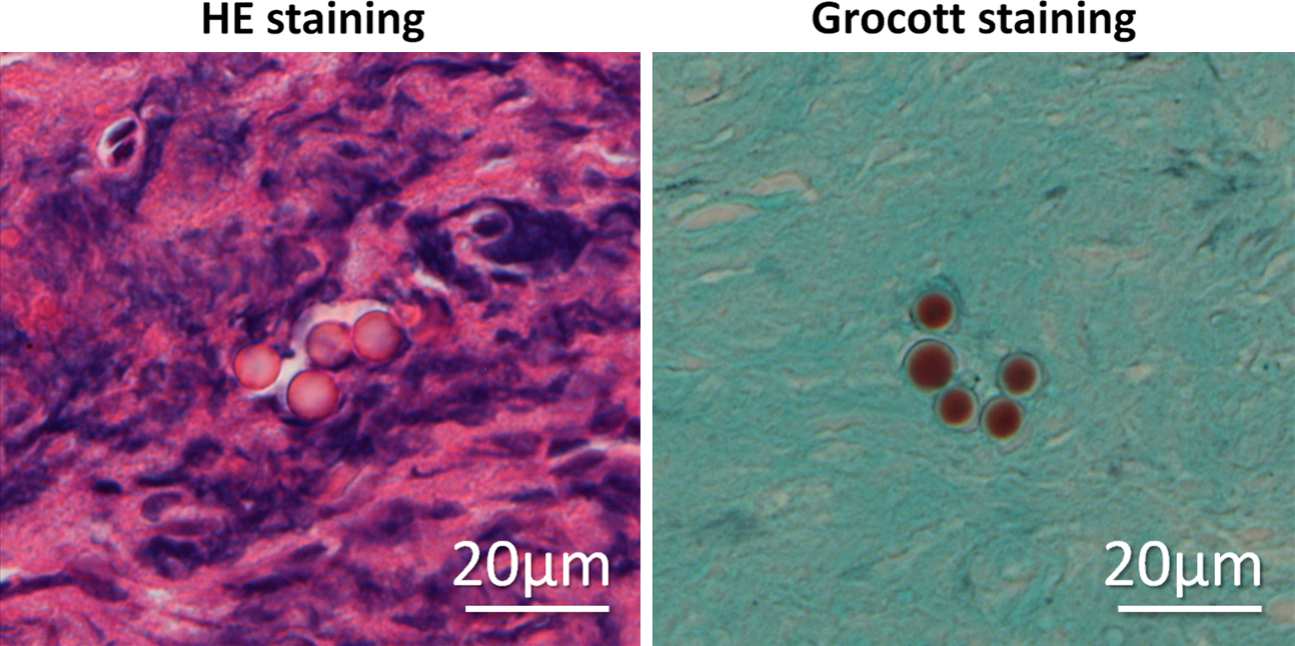
The encapsulated yeast in the extradural mass lesion. High magnification of HE staining shows a few spherical bodies, which have a large halo of unstained space surrounding each cell, representing capsules of *Cryptococcus neoformans* on Grocott staining

**Fig. 6:**
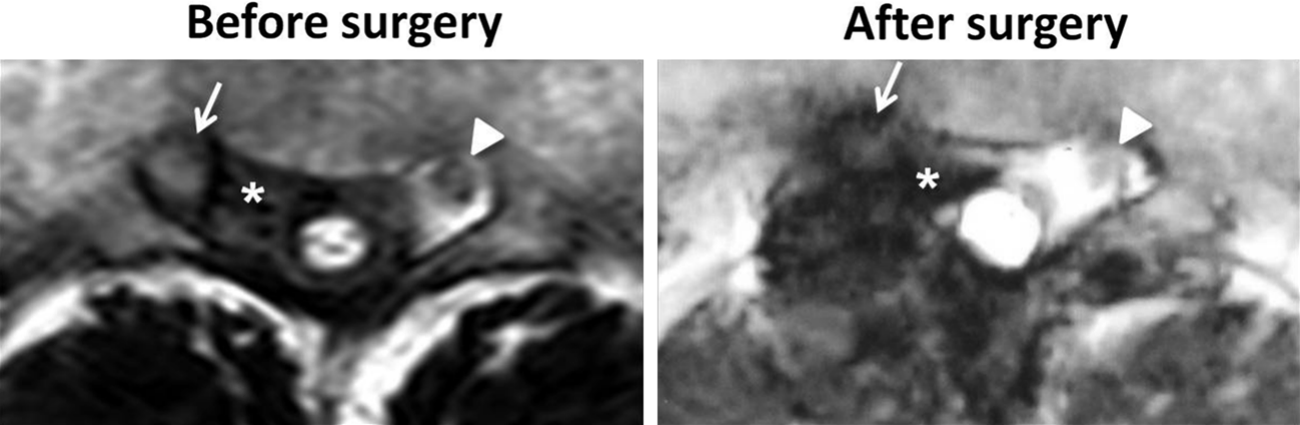
T2-weighted MRI of the sacral spine before and after surgery. The SOL (*asterisks*) seen before surgery is decreased and recognized only around the right S2 nerve root after surgery. *Arrows* and *arrowheads*, the *right* and *left* S2 nerve roots, respectively

Histological evaluation of the extradural lesion showed fibrous tissue with chronic inflammatory cell infiltrates, with no evidence of malignancy or hemosiderin deposits (Fig. 4a). The predominant cell type in the inflammatory focus was lymphocytes (Fig. 4b). Only a few refractile spherical bodies were found in the chronic inflammatory fibrous tissue on hematoxylin and eosin (HE) staining of the surgical specimen (Fig. 5), which suggested cryptococcal infection. Grocott staining showed the encapsulated yeasts (Fig. 5), and cryptococcal infection was diagnosed. The patient was then interviewed about his exposure history to avian species, and it was found that he had always kept 20–30 birds of several different kinds, such as pigeons, chickens, true parrots, and so on, from his childhood to his middle teens. Though a systemic examination was performed again, normal findings on X-ray and CT examinations of his chest were confirmed by a respiratory medicine specialist (co-author), and serum cryptococcal antigen testing was negative. Antifungal therapy was started with oral fluconazole (200 mg/day) after the definitive diagnosis was made. Since his symptoms still remained even 3 months after oral fluconazole was started, an intravenous infusion of liposomal amphotericin B (150 mg/day for 2 weeks) was substituted, followed by an intravenous infusion of fluconazole (200 mg/day for 2 weeks). Thereafter, oral itraconazole (100 mg/day) was administered for 4 months. T2-weighted imaging on spinal MRI 9 months after surgery showed that the SOL was significantly decreased, although a low intensity area was recognized around the right S2 nerve root (Fig. 6). The pain in his leg and perianal area almost subsided, and MRI has shown no change in his sacral spine area as of 2 years after surgery. The WBC count has remained high (11,310/μl), although the neutrophils have stayed in the normal range (65.9 %).

## Discussion

The CNS is the most frequent target of cryptococcal infection except for the lungs, because the soluble anticryptococcal factors present in serum are absent from CSF [17], and pulmonary cryptococcosis is not necessarily accompanied by a CNS lesion [18]. Involvement of the extradural space is a rare complication of cryptococcal nervous system infection, which is considered the result of a direct extension from the infected bone (calvaria or spine) or muscles [7–12, 14, 19]. In the present patient, the extradural infection did not follow contiguous regions such as skin, muscles, or bone, since these tissues revealed no abnormalities on both preoperative images (Figs. 1, 2) and intraoperative findings. Since the extradural fibrous lesion adhered firmly to the dura mater, the primary cryptococcal infection might have involved the meninges. In addition, the cauda equina in the dural sac was slightly enhanced by gadolinium on MRI, which suggests that meningoradiculitis might have accompanied the extradural infection. It has been reported that meningoradiculitis showed meningeal thickening and enhancement of the nerve root by gadolinium on MRI, although there was no extradural lesion in the previous papers [20, 21]. In the present case, the extradural fibrous lesion appeared quite massive, yet the intradural aspect seemed mild on MRI. Therefore, this suggested that cryptococcal infection might have occurred in the meninges and spread to the extradural space rather than to the intradural space. Considering that cryptococcal infection develops mainly in the subarachnoid space to cause meningitis [22], choroid plexitis [23], and encephalitis [18], massive spread to the extradural space, as in the present case, may be extremely rare.

Cryptococcoma is characterized by localized, solid, tumor-like masses with a chronic granulomatous reaction composed of macrophages, lymphocytes, and foreign body-type giant cells [24]. These lesions mostly appear in immunocompetent patients with a sufficient inflammatory response [24]. The cryptococcoma of the present patient showed a chronic inflammatory response consisting of numerous lymphocytes and fibrosis, which was thought to have formed over the years. Cryptococcal infection might have already started at a younger age, since he could have been constantly exposed to the yeasts in his childhood. There are two possibilities to explain why his high WBC count continued after surgical intervention or antifungal therapy: the cryptococcal infection had not completely subsided, or his high WBC count was unrelated to his cryptococcal infection.

Clinically, cryptococcal infection in the CNS may manifest itself in a variety of ways, such as encephalitis, tuberculous meningitis, general paresis, brain abscess, brain tumor, or spinal cord tumor. In particular, tuberculous meningitis resembles cryptococcal infection, not only clinically but also histologically, both of which are characterized by chronic inflammation, necrotic exudate, angiitis, and granulation tissue [25]. Definitive diagnosis of cryptococcal infection can only be established by microscopic identification of *Cryptococcus neoformans* or detection of cryptococcal antigen in the serum or CSF [2]. Unfortunately, the CSF was neither examined for cryptococcal antigen nor sent for culture in our case, since cryptococcal infection was not considered in the differential diagnosis of a healthy man without a respiratory lesion before surgery.

There is no standardized treatment protocol for cryptococcal infection of specific body sites, except for the lung and CNS. For cerebral cryptococcomas, surgery is recommended in the guidelines updated by the Infectious Disease Society of America in 2010 for large (≥3 cm), accessible lesions with a life-threatening mass effect [26]. Surgery may also be needed to achieve cure in cases unresponsive to repeated antifungal therapy, or partial removal followed by histopathology and culture are necessary for confirmation of diagnosis in cases indistinguishable from acute pyogenic abscesses or with symptomatic hydrocephalus [26]. The selection of antifungal agent and duration of therapy depends on factors that include the severity of disease, host immune status, the anatomic sites of involvement, and therapeutic response [26]. In Japan, treatment with 200–400 mg/day fluconazole for 3–6 months is recommended for pulmonary cryptococcosis without immunosuppressive risk factors in the Guidelines for Management of Deep-seated Mycoses 2007 [27]. Based on this guideline, the present patient was started on oral fluconazole at 200 mg/day. Although his symptoms were resistant to this initial therapy, intensified therapy was conducted with an intravenous infusion of liposomal amphotericin B and fluconazole. Antifungal therapy, both oral and intravenous, was continued for 8 months, and his symptoms subsided. There has been no remarkable change on the MRI of his sacral spine over these 2 years.

## Conclusion

Extradural cryptococcoma at the sacral spine with neither pulmonary cryptococcosis nor osteomyelitis in an immunocompetent individual is a rare condition that should be considered in the differential diagnosis of an extradural fibrous lesion in the spine.
